# The Bacterial Composition of the Gut Microbiota of Mexicans with Overweight and Obesity: A Systematic Review

**DOI:** 10.3390/microorganisms13081727

**Published:** 2025-07-24

**Authors:** María Alejandra Samudio-Cruz, Alexandra Luna-Angulo, Elizabeth Cabrera-Ruiz, Carlos Landa-Solis, Edgar Rangel-López, Paul Carrillo-Mora, Juan Ríos-Martínez, Rafael Toledo-Pérez, Rogelio Paniagua-Pérez, Carlos Jorge Martínez-Canseco, Ana Luisa Lino-González, Abigail Jhoana Saldaña Solano, Laura Sánchez-Chapul

**Affiliations:** 1 Clinical Neurosciences Division, National Institute of Rehabilitation “Luis Guillermo Ibarra Ibarra”, Mexico City 14389, Mexico; psic.alejandra.samudio@gmail.com (M.A.S.-C.); neuropolaco@yahoo.com.mx (P.C.-M.); ana_onil@yahoo.com.mx (A.L.L.-G.); 2 Neuromuscular Diseases Laboratory, Clinical Neurosciences Division, National Institute of Rehabilitation “Luis Guillermo Ibarra Ibarra”, Mexico City 14389, Mexico; lunangulo@gmail.com; 3 Basic Neurosciences Division, National Institute of Rehabilitation “Luis Guillermo Ibarra Ibarra”, Mexico City 14389, Mexico; elicabreraruiz@gmail.com; 4 Tissue Engineering, Cell Therapy and Regenerative Medicine Unit, National Institute of Rehabilitation “Luis Guillermo Ibarra Ibarra”, Mexico City 14389, Mexico; cls_73@hotmail.com; 5 Cell Reprogramming Laboratory, National Institute of Neurology and Neurosurgery “Manuel Velasco Suárez”, Mexico City 14269, Mexico; raledg@hotmail.com (E.R.-L.); abisaldannia@gmail.com (A.J.S.S.); 6 Instituto de Investigación en Ciencias de la Salud de la Secretaria de Marina, Mexico City 04849, Mexico; juan_rios_mtz@yahoo.com.mx (J.R.-M.); cencis.pr.rtoledo@uninav.edu.mx (R.T.-P.); 7 Servicio de Bioquímica, National Institute of Rehabilitation “Luis Guillermo Ibarra Ibarra”, Mexico City 14389, Mexico; rpaniagua@inr.gob.mx (R.P.-P.); cmartinez@inr.gob.mx (C.J.M.-C.)

**Keywords:** gut microbiota, dieta de la milpa, obesity, Mexican population, obese children, obese adults

## Abstract

The role of the gut microbiota in obesity has been extensively studied worldwide, but research in Mexican populations is still limited. This is particularly important given the high obesity rates in Mexico, despite a traditional diet rich in diverse, plant-based foods. We conducted a systematic review of studies examining the gut microbiota in obese Mexican children and adults. The literature search was conducted in the Medline, CINAHL, SciELO, Redalyc and Web of Science databases. The included studies addressed topics such as obesity in different Mexican subgroups (e.g., adults, children, rural communities), dietary behaviors and interventions, traditional dietary patterns, and gut microbiota composition. Of the 2332 datasets, 19 studies met the inclusion criteria. These studies indicated that obesity in Mexican individuals is associated with specific changes in the gut microbiota, including decreased bacterial diversity and shifts in the abundance of key microbial genera. Differences were found between age groups and regions. This review highlights a clear association between gut microbiota composition and obesity in the Mexican population. Further research is essential to investigate how the traditional Mexican diet may influence gut health and serve as a potential tool to treat obesity.

## 1. Introduction

Obesity is a major public health problem that has reached epidemic proportions and affects a growing number of children and adults worldwide [1]. According to the World Health Organization (WHO), around 1.9 billion adults were overweight in 2016, and 650 million of them were considered obese [2]. By 2022, 37 million children under the age of 5 will be overweight, while more than 390 million children and adolescents between the ages of 5 and 19 will be overweight, 160 million of whom will be diagnosed as obese [2]. In Mexico, although there are no precise and reliable official statistics, according to the National Health and Nutrition Survey [3] carried out in 2018, which included 16,257 adults over 20 years of age, 36% of adults have some degree of obesity (BMI > 30 kg/m^2^), finding a higher prevalence in women compared to men. Considering the numbers provided by these surveys between 2000 and 2018, a 42.2% increase in obesity rates has been observed in Mexico [4]. On the other hand, these national surveys observed a 19.6% obesity in school-aged children and 26.8% in adolescents in 2020, numbers that have also shown a continuous increase trend since 2006 [5].

This chronic disease has become a global challenge as its multifactorial etiology, such as genetics, hormones, unhealthy diet, and physical inactivity, contributes to an imbalance between energy expenditure and energy consumption [1], as well as an alteration in the composition of the gut microbiota (dysbiosis) [6,7]. This dysbiosis increases the energy yield from food, alters peripheral metabolism, and increases body weight [8,9], which has an impact on physical and mental health [10].

The gut microbiota is a dynamic ecosystem that colonizes the human intestinal tract and consists of trillions of microorganisms, mainly dominated by bacteria, including protozoa, viruses, archaea, and fungi [11]. In a healthy state, the gut microbiota has specific and crucial functions that influence the normal physiology and disease susceptibility of the host [12], prevent the growth of pathogenic bacteria, maintain immune homeostasis, influence digestion and absorption of essential nutrients, and regulate metabolic processes [13]. However, the intestinal microenvironment of the host has an important influence on the structure and function of the microorganisms that inhabit it [14].

The composition of the gut microbiota varies from person to person and depends on age, dietary habits, and environmental factors [6,7]. Most microorganisms belong to the phyla *Bacillota* (formerly known as *Firmicutes*), *Bacteroidota*, *Actinobacteria*, and *Proteobacteria* [6,7]. The *Bacillota* are the largest bacterial phylum and comprise more than 200 genera, including *Lactobacillus*, *Mycoplasma*, *Bacillus*, and *Clostridium*. The *Bacteroidota* include the genus *Bacteroides* (restricted to species of the *Bacteroides fragilis* group), the *Actinobacteria* include the genus *Bifidobacterium* (considered probiotic) and the *Pseudomonadota* (formerly known as *Proteobacteria*) include a variety of pathogenic bacteria such as enterotoxigenic *Escherichia coli*, enteroinvasive *Escherichia coli*, enteropathogenic *Escherichia coli*, and enteroaggregative *Escherichia coli* [9]. Early studies on the gut microbiota suggested that the ratio of *Bacillota*/*Bacteroidota* was higher in obese individuals [15]. However, later studies did not confirm this finding, as no differences were found in the proportions of *Bacteroidota* and *Bacillota* in the feces of lean and obese individuals [16].

Diet and exercise also play a crucial role in modulating the gut microbiota [17,18]. Healthy diets such as the Mediterranean diet, which is rich in fruits, vegetables, fiber and omega-3 fatty acids [19], and the traditional Mexican diet, which is based on traditional foods such as “La dieta de la Milpa” (cornfield diet) [20], have a positive effect on the composition of the gut microbiota; they increase the number of beneficial bacteria such as *Fecalibacterium prausnitzii*, *Roseburia*, *Ruminococcus*, *Bacteroides*, *Bifidobacteria* and *Lactobacilli*, which can contribute to the prevention and treatment of obesity [19,20] and improve variables such as BMI, lipids, and blood glucose levels [20]. Regular exercise modulates the human gut microbiota and promotes the growth of beneficial bacteria [21]. However, further research is needed to better understand the mechanisms and provide guidelines for the type and intensity of exercise.

Research on the gut microbiota of people with obesity in Mexico is still limited compared to studies from other countries such as North America, Europe, and East Asia. However, previous findings suggest that obese Mexicans have lower microbial richness and diversity than normal weight individuals and that the composition of microbial groups is pro-inflammatory and metabolically detrimental [22,23]. Given the importance of the gut microbiota in obesity, the high prevalence of this disease in Mexico, and the rich and diverse plant-based dietary traditions, we conducted a systematic review of recent interdisciplinary contributions by Mexican research teams that have investigated the role of the gut microbiota in obesity, emphasizing the bacterial composition observed in this population.

## 2. Materials and Methods

### 2.1. Study Design and Searches

This systematic review was conducted following the PRISMA (Preferred Reporting Items for Systematic Reviews and Meta-Analyses) guidelines [24] and duly registered with the PROSPERO International Prospective Register of Systematic Reviews (Registration ID: CRD420251046664). A comprehensive literature search was conducted in the Medline, CINAHL, SciELO, Redalyc and Web of Science databases, using a combination of the two connectors “AND” or “OR” to identify relevant studies on the main research topics, including “Mexican humans with obesity”, “Mexican adults”, “Mexican children”, “Mexican women”, “rural communities in Mexico”, “Mexican schoolchildren”, rural Mexico, “young Mexican adults”, “obesity”, “adult obesity”, “childhood obesity”, “pediatric obesity”, “dietary patterns”, “dietary interventions”, “Mexican food”, “traditional Mexican diet”, “Mexican diet”, “food consumption”, “human gut microbiota”, “gut microbio”, “microbio”, ”gut bacteria”, “gut composition”, “Intestinal bacteria”, and “gut commensals”. All references found were imported into EndNote Reference Manager for data management. Duplicate entries were identified and removed both automatically via software (Rayyan; https://rayyan.ai/cite, accessed on 21 July 2025) and manually by two independent reviewers. In addition, the reference lists of all included studies were checked to identify further suitable sources. The search was restricted to articles published up to 31 December 2024.

### 2.2. Eligibility Criteria and Study Selection

**Figure 1:**
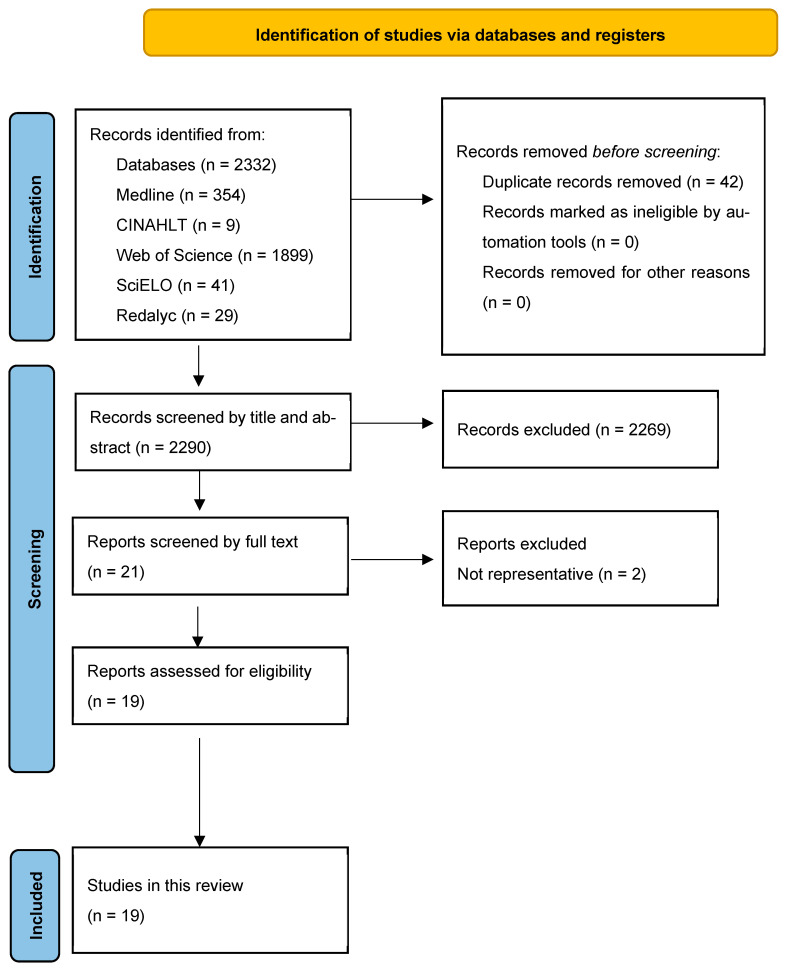
Flow diagram of the study selection process.

Relevant publications were selected through an initial screening of titles and abstracts by two independent reviewers, who excluded studies that did not meet the eligibility criteria. Studies were included if they met the following criteria: (A) Original cross-sectional, longitudinal, and interventional studies characterizing the gut microbiota of Mexicans with obesity and (B) availability of full-text articles published in English and Spanish. Exclusion criteria were as follows: (A) Systematic reviews or meta-analyses studies, letters to the editor or articles that only publish the research protocols without presenting the results and discussion section, (B) experimental research with animals, and (C) gray literature (congress or conference material, guidelines, notes, abstracts). The study selection process is illustrated in the PRISMA flowchart (Figure 1).

### 2.3. Data Extraction

The full-text articles of studies deemed potentially eligible based on the inclusion criteria were thoroughly screened, and the relevant data were extracted independently by two reviewers and compiled in a pre-specified data extraction table. The extracted data included the following variables: author, date, design, sample size, participants’ characteristics, regions of Mexico such as central, western, eastern and southern Mexico, dietary components, microbiota assessment method, and outcome.

### 2.4. Quality Assessment

The quality of the included manuscripts was assessed using the Critical Appraisal tools for use in JBI Systematic Reviews for analytical cross-sectional studies, quasi-experimental studies, and randomized controlled trials. The maximum score attainable is 8 points for analytical cross-sectional studies, 9 points for quasi-experimental studies, and 13 points for randomized controlled trials.

## 3. Results

### 3.1. Searches and Study Selection

The search initially identified a total of 2332 articles. After removing duplicates and articles that did not meet the eligibility criteria, 2290 articles were screened. Of these, 2269 were excluded because they were review articles, animal studies, or not relevant to the main topic. Of the 21 full-text articles assessed, those that lacked relevant or sufficient results or did not represent the target population were excluded. Finally, 19 studies were included in this systematic review [23,25,26,27,28,29,30,31,32,33,34,35,36,37,38,39,40,41,42]. A detailed flowchart of the study selection process is shown in Figure 1, and the characteristics of the 19 included studies are listed in Table 1.

### 3.2. Quality Assessment

Of the 19 studies included in this review, 17 were cross-sectional studies, one was a randomized clinical trial and one was a quasi-experimental pre-post study. Of the cross-sectional studies, 13 achieved the maximum quality score (8 points), three scored 6 points and one scored 5 points. The clinical trial received 6 out of 9 possible points, while the pre-post study scored 6 out of 13 points. Although some studies received a relatively low-quality score, we chose to include them due to the limited availability of literature on this topic. The result of the quality assessment of the articles is shown in the Supplementary Table S2.

### 3.3. Data Extraction

All studies included in this review were published between 2015 and 2024 and were conducted in Mexico. Sample sizes varied widely, ranging from 6 to 1042 participants. Research designs included cross-sectional, analytic, descriptive, and transversal studies, as well as short-term quasi-experimental longitudinal pilot studies and double-blind trials. The age of the participants ranged from 6 to 50 years. Various analytical techniques were used to assess the gut microbiota: 16S rRNA gene sequencing was the most common, while three studies used species-specific qualitative PCR. Other methods included high-throughput DNA sequencing, 16S rRNA amplicon shotgun sequencing, metagenomic shotgun sequencing, and ion-torrent semiconductor sequencing of 16S rDNA. Obesity was diagnosed using several indices: body mass index (BMI), body composition (including body fat and muscle mass percentage), waist circumference (WC), and hip circumference (HC).

**Table 1:** Characteristics of the included studies that investigated the composition of the gut microbiota of Mexicans with obesity.

	Author	Date	Design	Sample Size	Participant’s Characteristics	Region of Mexico	Dietary Components	Microbiota Assessment Method	Outcome
1	Laura Moreno-Altamirano [36]	2024	Cross-sectional study	91	Medical students aged 18 years or older (65 women and 26 males) divided according to BMI into normal weight and overweight/obese	Central Mexico (Mexico City)	Specified in the food frequency questionnaire (FFQ)	16S rRNA gene sequencing	*Bacteroides* and *Prevotella* were the predominant genera across different BMI categories. *Bacteroides* was more prevalent among men with overweight + obesity, while *Prevotella* was more common in men with normal weight. These trends were not observed in women.
2	Verónica Riggen-Bueno [40]	2024	Cross-sectional, comparative study	65	Volunteer male and female participants aged between 18 and 59 years were divided according to BMI into normal weight control and overweight/obese	Western Mexican states (Colima, Jalisco, Michoacán, and Nayarit)	The FFQ was not applied	16S rRNA gene sequencing	The gut microbiota of the obese group showed a notable increase in *Negativicutes*, *Escherichia/Shigella*, *Prevotella*, and *Lachnoclostridium.*
3	Ricardo García-Gamboa [29]	2024	Cross-sectional study	30	Men and women aged between 20 and 50 years	NS	Specified in the FFQ and in the 24 h dietary recall	16S rRNA gene sequencing	The *Bacillota/Bacteroidota* and *Bacteroides/Prevotella* ratios were positively associated with BMI. In overweight and obese individuals, those with higher levels of *Akkermansia muciniphila* had more favorable lipid and glucose profiles than those with lower levels of this bacterium. Obese subjects had *Allisonella*, *Subdoligranulum* and *Dielma*, *Lachnospira*, *Romboutsia*, and *Clostridium*. Overweight individuals had *Flavonifractor*, *Eggerthella*, and *Alloprevotella.* Healthy weight individuals had *Faecalibacterium*, *Histophilus*, *Rikenella*, *Odoribacter*, and *Marvinbryantia*.
4	Avilene Rodríguez-Lara [41]	2022	Cross-sectional study	120	Mexican students (females and males) aged between 18 and 25 years were divided according to BMI into normal weight and overweight/obese	Western Mexico (Guadalajara, Jalisco)	Specified in the FFQ	Species-specific qualitative PCR	The phyla *Bacillota* with mainly *Clostridium coccoides-Eubacterium rectale* were found to be mostly expressed in overweight and obese individuals compared to the normal weight subjects.
5	Sofía Morán-Ramos [35]	2022	Short-term, quasi-experimental, longitudinal pilot study. Open, self-controlled clinical trial	6	Male children aged 12 years. Obesity status was defined according to the BMI score. Intervention of 6 weeks of diet and exercise	Central Mexico (Mexico City)	Dietary information was obtained. Intervention: participants received a hypo energetic dietary plan (60% energy from carbohydrates, 20–25% from fats, and 15–20% from proteins)	16S rRNA gene sequencing	The most abundant *phylum* in children before the intervention was *Bacteroidota*, *Bacillota* and *Proteobacteria*, and no significant modifications in this abundance were observed after the intervention. *Odoribacter* was associated with the reduction in waist circumference after the intervention.
6	Marco U. Martinez-Martinez [30]	2022	Controlled clinical trial. Randomized double-blind	38	Male and female children aged between 6 and 10 years. Obesity status was defined according to the BMI percentile for overweight and obesity	Eastern Mexico (Escalerillas, San Luis Potosí)	All children had a similar diet at the full-time school program.	16S rRNA gene sequencing	In children with overweight or obesity *Veillonella*, *Catenibacterium*, *Blautia*, *Alistipes* and *Holdemanella* showed a positive association with the BMI and BMI Z score. A synbiotic with fructans from *Agave salmiana* stimulated the abundance and diversity of genera *Faecalibacterium* and *Holdemanella*, thus, leading to a healthier gut microbiota.
7	Miguel Vázquez-Moreno [42]	2020	Cross-sectional analytical observational study (with case-control statistical analysis)	330	Unrelated children with normal weight and obesity aged 6–12 years. Children with a BMI ≥ 5th and <85th percentile were classified as having normal weight and those with a BMI ≥ 95th percentile as having obesity	Central Mexico (Mexico City) Southern Mexico (Oaxaca)	The FFQ was not applied	16S rRNA amplicon sequencing	*Fusicatenibacter*, *Romboutsia*, *Ruminococcaceae Ruminiclostridium*, *Blautia*, *Clostridium*, *Anaerostipes* and *Intestinibacter* were associated with obesity. In Mexico City, the most relative abundant bacteria in all the samples were *Blautia*, *Ruminococcaceae* UCG-002, and *Anaerostipes.* Obesity status was positively associated with *Fusicatenibacter* and *Romboutsia*, and negatively associated with *Ruminococcaceae* UCG-002 and *Ruminiclostridium*. In Oaxaca, the most abundant bacteria were *Bacteroides*, *Alistipes*, and *Clostridium*_*sensu_stricto*_1. No genera were found to be associated with obesity status.
8	Tania Aguilar [25]	2020	Cross-sectional study	93	Normal weight, overweight, and obese school-aged children aged 8.4 ± 1.6 years (girls and boys)	Eastern Mexico (rural communities (Santa Maria Begoña and Santa Cruz) of Queretaro	The FFQ was not applied	Species-specific qualitative PCR	Lower *Bacteroidaceae–Porphyromonadaceae–Prevotellaceae* and higher abundance of *Lactobacillaceae* were associated with obesity and metabolic disturbances.
9	Luigui Gallardo-Becerra [28]	2020	Cross-sectional study	27	Children (girls and boys) aged 7–10 years: normal weight (NW), obese (O), and obese with metabolic syndrome (OMS) children from a summer-camp of Mexican Health Ministry employees. All children came from households with a middle economic class income and belonged to a similar socio-cultural status. Obesity was defined by body mass index (BMI) based on the guidelines of the Centers for Disease Control and Prevention (CDC).	Central Mexico (Mexico City)	The FFQ was not applied	Sequencing shotgun of the total RNA and a profiling of the 16S rRNA gene using the V4 region	*Bacillota* were markedly increased in the OMS and O compared to the NW group. *Bacteroidota* were increased in NW compared to the O and OMS. Novel biomarkers for obesity with MetS consisted of an increased *Coriobacteraceae*, *Collinsela*, and *Collinsella aerofaciens*; *Erysipelotrichaceae*, *Catenibacterium* and *Catenibacterium* spp., and decreased *Parabacteroides distasonis*, which correlated with clinical and anthropometric parameters associated to obesity and metabolic syndrome. Genus *Porphyromonas* and an undetermined species within this genus were specifically over-abundant in the obese group.
10	Sofía Morán-Ramos [34]	2020	Cross-sectional study	926	School children aged 6–12 years. Obesity status was defined as BMI percentile	Central Mexico (Mexico City)	Specified in the FFQ	16S rRNA amplicon sequencing	63% of the children belonged to *Bacteroides* enterotype, while 37% belonged to *Prevotella* enterotype. The more abundant taxa were *Bacteroides* followed by *Prevotella* and an unclassified *Ruminococcaceae* genus. Greater abundance of *Christensenellaceae* family was associated with a lower BMI percentile.
11	Alejandra Chávez-Carbajal [26]	2019	Cross-sectional analytic study	64	Volunteer healthy Mexican women (CO), women with obesity (OB), and women with obesity plus metabolic syndrome (OMS) aged from 18 to 59 years	Central Mexico (Mexico City)	Specified in the FFQ	Species-specific qualitative PCR	*Bacillota* were more abundant in women with OB or OMS than in women of the CO group. There were significant changes in abundances of bacteria belonging to the *Ruminococcaceae*, *Lachnospiraceae*, and *Erysipelotrichaceae* families. The *Proteobacteria*, *Actinobacteria*, *Tenericutes*, *Cyanobacteria*, and *Synergistetes* were also not different between the groups. The “Others” category, which included less abundant phyla such as *Verrucomicrobia*, *Spirochaetes*, and *Fusobacteria*, showed a significant difference between groups. *Faecalibacterium* spp., *Roseburia* spp., *Lachnospira* spp., and *Coprococcus* spp. were significantly more abundant in the OB and OMS groups. The family *Erysipelotrichaceae* was significantly decreased in the OB and OMS groups.
12	Otoniel Maya-Lucas [31]	2019	Cross-sectional study	20	Unrelated children aged between 9 and 11 years selected from a 118 obesity database	Central Mexico (Mexico City)	Specified in the FFQ	Metagenomic shotgun-sequencing of DNA	In obese children, there was an increase in unclassified *Methanobrevibacter* spp. Normal weight children had a community dominated by *Ruminococcus* spp. (enterotype 3). Obese children had a community dominated by *Prevotella* spp. (enterotype 2), *Megamonas* spp. were overrepresented, and members of the family *Oscillospiraceae* were depleted.
13	Khemlal Nirmalkar [38]	2018	Cross-sectional study	172	Children aged 6–11 years and adolescents aged 12–18 years. Obesity was defined according to BMI percentile based on Centers for Disease Control and Prevention (CDC) reference data.	Central Mexico (Toluca, State of Mexico)	Diet intake was divided into seven food groups as follows: (1) Starchy staples, (2) legumes, (3) dairy, (4) meat, (5) vitamin A-rich fruits and vegetables, (6) other fruits and vegetables or fruit juices, and (7) foods made with oil, fat, or butter.	16S rRNA amplicon sequencing	Obese children and adolescents showed an increase in the abundance of members of the family *Coriobacteriaceae* (*p-Actinobacteria*). The genus *Lactobacillus* and family *Coriobacteriaceae* were enriched in children, and genera *Collinsella* and *Prevotella* were enriched in obese adolescents.
14	Berenice Lopez-Contreras [23]	2018	Cross-sectional study	138	Normal weight and obese children aged 6–12 years from a summer camp for children of Mexican Health Ministry employees. Obesity was defined according to BMI percentile based on Centers for Disease Control and Prevention (CDC) reference data.	Central Mexico (Mexico City)	Specified in the FFQ	16S rRNA amplicon sequencing	Normal weight and obese children had no significant differences in phyla abundances or *Bacillota/Bacteroidota* ratios. In obese children, *Bacteroides eggerthii* abundance was significantly high and correlated positively with body fat percentage. In normal weight children, *Bacteroides plebeius* and unclassified *Christensenellaceae* abundances were significantly higher.
15	Eder Orlando Méndez-Salazar [33]	2018	Cross-sectional study	36	Mexican schooled children aged 9–11 years belonging to low-income families	Central Mexico (Chimalhuacán, State of Mexico)	Specified in an unannounced 24 h dietary recalls.	16S rRNA gene sequencing	Undernourished children had significantly higher levels of bacteria in the *Bacillota* phylum and in the *Lachnospiraceae* family than obese children, while the *Proteobacteria* phylum was overrepresented in the obese group.
16	Yaneth C Orbe-Orihuela [39]	2018	Cross-sectional study	890	Normal weight, overweight, and obese children residents aged from 6 to 14 years	Central Mexico (Mexico City)	Specified in the FFQ	Species-specific qualitative PCR	High relative abundance of *Bacillota* with high *Bacillota/Bacteroidota* ratio was found in obese children
17	Lino Mayorga Reyes [32]	2016	Cross-sectional study	9	Young adults women and men aged from 18 to 39 years from the Universidad Autónoma Metropolitana classified as lean, overweight, and obese	Central Mexico (Mexico City)	Specified in the FFQ	Species-specific qualitative PCR	There were significant differences in the gut microbiota between the overweight and lean groups. There were no significant differences in the abundance of the *Bacillota* and *Bacteroidota* within each group. Striking significant differences were observed in the abundance of *Faecalibacterium prausnitzii* between lean and obese groups and between overweight and obese groups. There were significant differences in the abundance of *Clostridium leptum* between lean and obese groups and between overweight and obese groups. For *Bifidobacterium longum*, significant differences were found between lean and overweight groups and between lean and obese groups. Members of the *Bacillota* phylum and *B. longum* were more abundant in the lean group.
18	Barbara Ixchel Estrada-Velasco [27]	2014	Cross-sectional study	1042	Unrelated children aged 6–14 years	Mexico City (North, South, East and West)	Dietary patterns: Mexican snacks and fast food, fresh fruits and vegetables, fish and foods with saturated fat, tortillas and corn-based foods, bread, cereals, potatoes, rice, pasta, red meat, sausages	Species-specific qualitative PCR	High relative abundance of *Bacillota* and a low relative abundance of *Bacteroidota* were associated to obesity
19	S. Murugesan [37]	2015	Cross-sectional study	190	Unrelated children aged 9–11 years (normal, overweight, and obese). Obesity was defined according to BMI percentile.	Central Mexico (Ecatepec, State of Mexico)	Specified in a 7-day recall	Ion torrent semiconductor sequencing of 16S rDNA	Overweight and obese children presented an increased abundance of *Faecalibacterium* spp., *Lachnospiraceae*, and *Roseburia* spp.

NS: not specified.

In adults and children, several microbial taxa have shown changes associated with obesity indicators. In children, obesity-related microbial changes associated with obesity indicators have been observed in a broader range of taxa such as *Bacteroides*, *Bacillota*, *Bacteroidota*, *Proteobacteria*, *Veillonella*, *Catenibacterium*, *Blautia*, *Alistipes*, *Holdemanella*, *Fusicatenibacter*, *Romboutsia*, *Ruminococcaceae UCG-002*, *Ruminiclostridium*, *Clostridium*, *Anaerostipes*, *Intestinibacter*, and members of the families *Bacteroidaceae*, *Porphyromonadaceae*, *Prevotellaceae*, *Lactobacillaceae*, *Lachnospiraceae*, *Coriobacteraceae*, *Erysipelotrichaceae*, *Christensenellaceae*, and *Oscillospiraceae*. Specific taxa such as *Collinsella aerofaciens*, *Parabacteroides distasonis*, *Bacteroides eggerthii*, *Bifidobacterium longum*, *Faecalibacterium* sp., and *Roseburia*, as well as *Methanobrevibacter* spp. and *Megamonas* spp., were also involved [23,25,27,28,30,31,32,33,35,37,38].

In adults, *Bacteroides*, *Prevotella*, members of the class *Negativicutes*, *Escherichia*/*Shigella*, *Lachnoclostridium*, and taxa from the phyla *Bacillota* and *Bacteroidota* were detected. Other groups affected included *Clostridium coccoides*, the *Eubacterium rectale* group, families such as *Ruminococcaceae*, *Lachnospiraceae*, and *Erysipelotrichaceae*, as well as the phyla *Verrucomicrobia*, *Spirochaetes*, *Fusobacteria*, and *Faecalibacterium* spp. [26,29,36,40,41].

**Figure 2:**
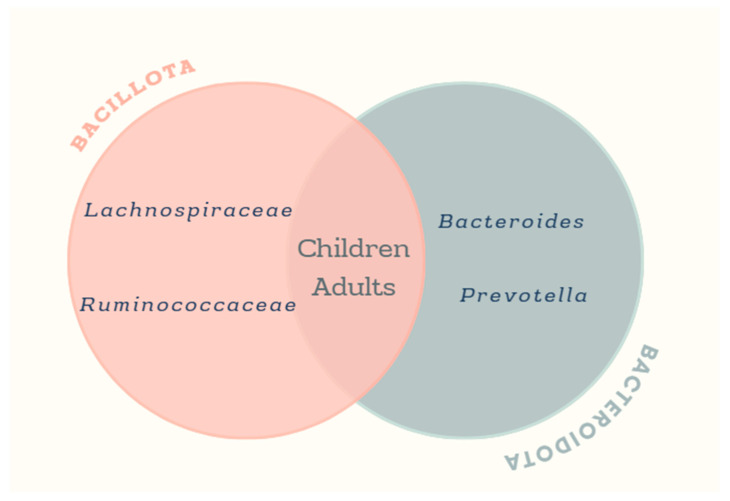
Overlap in bacterial signature between Mexican children and adults.

Although some taxa of the microbial signature of obesity in adults and children overlap, their complexity and composition may reflect an age-dependent interaction between the host and microbes (Figure 2).

### 3.4. Studies of the Gut Microbiota of Obese and Overweight Mexican Children

Overweight and obesity in children and adolescents have become one of the most important public health problems worldwide due to their high prevalence and the multiple metabolic, cardiovascular, and psychosocial complications they entail. The Mexican population has been subjected to different socioeconomic, agricultural, and demographic projects for decades, which have affected public health in different regions of the country and, consequently, the ease of food consumption in children and adults, as reported by their nutritional status [3,43,44]. Obesity is a nutritional problem in which dietary habits play a noticeable role in the composition of the microbiome [45]. In Mexico, approximately 34% of children between the ages of 5 and 11 are classified as overweight or obese [46,47]. Malnutrition and an excess of calories from a minimal food intake have been associated with overweight, obesity, type 2 diabetes mellitus, and metabolic syndrome (MetS), which are major contributors to these health threats [21]. These alarming data reflect a growing public health problem and emphasize the need to explore the causes and possible interventions [48]. Childhood obesity is the result of factors involved in its development, such as low or excessive birth weight, rapid growth after birth, high protein intake in the first months of life, lack of or short breastfeeding, consumption of energy-dense foods, and physical inactivity [3,49]. Dyslipidemia and type 2 diabetes have also been associated with obesity in infants [23,50]. On the other hand, the gut microbiome has been shown to play a crucial role not only in the production of microbial metabolites but also in the regulation of energy metabolism and host homeostasis, and it has been recognized as a potential trigger for metabolic changes in children that could develop into obese adults [51,52,53]. The studies on the gut microbiota of Mexican children were only conducted in three regions of Mexico: the center, the east, and the south. We found that certain taxa overlap in central and eastern Mexico, except in southern Mexico, where *Bacteroides* and *Alistipes* were found only in Oaxaca (Table 2).

The study of a cohort of approximately 1000 Mexican school children from Mexico City identified environmental variables and intrinsic host factors associated with the development of overweight, obesity, and MetS, suggesting that maturation of microbial diversity is related to age [34]. More than 40% of participants in this cohort were categorized as overweight or obese based on BMI. DNA sequence analysis of stool samples from these individuals revealed that the major bacterial phyla in the stool were *Bacillota* and *Bacteroidota*, with *Bacteroides* being the most abundant genus, followed by *Prevotella* and *Ruminococcace*. These data suggest that gut dysbiosis is not only dependent on bacterial composition, but also on age and socioeconomic status, including dietary habits [34] and metabolic abnormalities related to higher serum branched-chain amino acid (BCAA), aromatic amino acid (AA), arginine and proline concentrations, which are correlated with lower richness and bacterial diversity [34].

Another study of Mexican children from Mexico City aged 6 to 12 years also found that physical activity and dietary interventions in this population resulted not only in a reduction in WC but also in a significant increase in fecal *Odoribacter* levels, even when other anthropometric markers of obesity (such as weight, height, BMI and body fat composition) as well as gut microbiome composition and bacterial diversity remained unchanged [54]. *Odoribacter* belongs to a new taxon within the *Bacteroidota* and is a common short-chain fatty acid (SCFA)-producing member of the human gut microbiome. Reduced abundance of *Odoribacter* has been associated with various microbiologically induced diseases [53,55].

The composition of the gut microbiota in Mexican children aged 6 to 12 years from Mexico City, who were categorized into obese and normal weight according to BMI, showed an interesting association with amino acid serum levels and some metabolic disorders related to obesity. In this study, no significant differences were found between the bacterial phylum or in the *Bacillota*/*Bacteroidota* ratio between obese participants and normal weight children [23]. However, the relative abundance of *Bacteroides eggerthii* was higher in obese children and correlated positively with body fat percentage in this group. In contrast, *Bacteroides plebeius* and the *Christensenellaceae* family were more abundant in normal weight children, and their abundance was inversely correlated with serum phenylalanine levels, which have been associated with obesity and changes in adiposity in Mexican children [23]. Notably, the relative abundance of some bacterial species is influenced by the consumption of high levels of aromatic and branched-chain amino acids in the diet, and both factors have been strongly associated with obesity and diabetes [56]. Furthermore, the increased abundance of these bacterial species correlated with higher serum levels of amino acids such as valine, leucine/isoleucine, phenylalanine, and tyrosine in obese children. In particular, serum levels of phenylalanine correlated negatively with the abundance of *B. plebeius* and the *Christensenellaceae* family [23].

A study conducted on overweight and obese unrelated Mexican children aged 5 to 11 years from Ecatepec, State of Mexico, classified according to their BMI, showed that SCFA levels were elevated in these children, as evidenced by chemoanalytical tests that revealed high butyrate and propionic acid concentrations in their feces. In addition, a slight decrease in the abundance of the *Proteobacteria* phylum and some increase in the abundance of *Bacillota* were found in stool samples from obese children compared to normal weight children [37]. In the same context, a cross-sectional study of more than 1000 Mexican children aged 6 to 14 years from different regions of Mexico City (north, south, east, and west) showed that obesity was associated not only with a high-fat and high-carbohydrate diet, but also with a high proportion of *Bacillota* but a low proportion of *Bacteroidota* in the stools of obese and overweight children compared with normal weight individuals from the same areas of Mexico City [27]. In contrast, a study of the gut microbiota of children aged 9 to 11 years who were categorized as obese based on their BMI showed higher levels of *Proteobacteria* in stool samples compared to undernourished children from low-income families from Chimalhuacán, State of Mexico, with a positive correlation between these bacteria and fat intake in the obese group, while undernourished children had higher levels of *Bacillota* and the *Lachnospiraceae* family compared to obese individuals from Mexico City [33]. These studies highlight the importance of conducting more detailed studies to determine whether the level of *Proteobacteria* in stool samples could be a potential marker for the development of obesity [57].

A study of Mexican children aged 9 to 11 years showed that the levels of these SCFAs are elevated in obese children compared to normal weight individuals, suggesting that these elevations affect the cholesterol biosynthesis pathway in the organism [31]. This study found that obese children have lower levels of bacteria from the *Oscillospiraceae* family, which correlate negatively with serum total cholesterol levels. This is in contrast to normal weight children, whose gut microbiome is characterized by a higher abundance of these bacteria. Here, it was also shown that the relative abundance of *Methanobrevibacter* spp., *Megamonas* spp., and *Prevotella* spp. (enterotype 2) was significantly increased in obese children, whereas in normal weight children, the bacterial composition of the gut was dominated by *Ruminococcus* spp. (enterotype 3) [58]. Interestingly, the results of the metagenomic studies showed no significant differences in the composition of the global gut microbiome in obese Mexican children compared to normal weight children [23,31].

Recently, it was shown that weight, height, and waist/ hip circumference were higher in Mexican overweight/obese children (507 participants) than in healthy children (383 subjects), all under 14 years of age and from Mexico City [39]. Interestingly, the authors found that the levels of TNF-α and IL-6 (pro-inflammatory cytokines) were higher in the overweight/obese children group, while the levels of IL-10 (anti-inflammatory cytokine) were lower compared to the healthy children. In addition, the relative abundance of *Bacillota* was increased in the overweight/obese children group, while the levels of *Bacteroidota* were decreased. The authors hypothesized that persistent childhood obesity is an important factor in the development of a chronic imbalance between pro- and anti-inflammatory cytokines in the organism, such as TNF-α and IL-6, which are actively overexpressed in obese children [39]. In addition, endothelial dysfunction has been associated with the aforementioned increased secretion of the pro-inflammatory cytokines TNFa and IL-1 and the expression of some adhesion molecules in endothelial cells that occur in obesity [59]. Recently, the association between gut microbiota diversity and the expression of markers of endothelial dysfunction was demonstrated in obese Mexican children and adolescents aged 6–11 years and 12–18 years, respectively, from Toluca, State of Mexico [38]. In this study, anthropometric measures, clinical parameters, metabolic factors, and some markers of endothelial dysfunction were determined. The study showed a significant correlation between the expression of vascular cell adhesion molecule 1 (VCAM-1) and the bacterial *Veillonellaceae* family in obese children. The expression of ICAM-1 also showed a positive association with the abundance of *Ruminococcus* in obese children. In obese adolescents, the abundance of *Bacteroides* spp. was positively associated with the expression of ICAM-1. Similarly, total cholesterol levels showed a positive association with the abundance of *Ruminococcus*. In addition, the molecular approach to determine the relative abundance of bacterial taxa showed that the genus *Lactobacillus* and the family *Coriobacteriaceae* were more abundant in children, while the genera *Collinsella* and *Prevotella* were enriched in obese adolescents [38].

Aguilar et al. (2020) also showed that the relative abundance of certain bacterial families in the composition of the gut microbiota is closely associated with disturbances in metabolic markers and obesity in Mexican children [25]. The authors found that obese children had lower levels of *Bacteroidaceae–Porphyromonadaceae–Prevotellaceae*, while the relative abundance of *Lactobacillaceae* was higher compared to healthy children. Similar to previous reports, obese children were found to have higher TNF-α levels and lower IL-10 levels. This inflammatory profile could influence intestinal permeability and induce the inflammatory state observed in obesity and insulin resistance [60]. The authors therefore hypothesize that lower levels of *Bacteroidaceae–Porphyromonadaceae–Prevotellaceae* may represent a link between obesity and some metabolic disorders [25].

The composition of the secretome of the gut microbiome influences its composition by affecting the growth and persistence of certain bacterial populations and thus their interactions with the host’s immune system through the modulation of inflammatory responses. Recently, a study used a metatranscriptomic approach to characterize the secretome produced for the gut microbiota in a cohort of Mexican school children (approximately 9 years) of similar socioeconomic and cultural status from Mexico City [28]. These participants were categorized into obese participants, obese children with MetS, and healthy children based on anthropometric measures such as BMI, WC, age, and gender. Taxonomic analysis of the stool samples showed that the most common phyla in the groups studied were *Bacillota*, *Bacteroidota*, and *Proteobacteria*. *Bacillota*, in particular, were significantly more common in the groups of obese and overweight children compared to the normal weight children. On the other hand, the genus *Porphyromonas* was overrepresented in the obese group, and the authors propose this genus as a potential biomarker for obesity. In addition, the genus *Collinsella* and its related species *Collinsella aerofaciens* were significantly more prevalent in the obese children with MetS group. Interestingly, the relative abundance of *Parabacteroides distasonis* was reduced in the same group. Therefore, these bacteria could be considered as potential markers for obese patients with metabolic disorders [28]. In addition, *C. aerofaciens* and *Faecalibacterium prausnitzii* are associated with clinical parameters such as increased triglycerides (TGs) and decreased HDL cholesterol [28], which have important metabolic implications, because low HDL cholesterol levels correlate independently and inversely with the presence of cardiovascular disease and impair the functions of HDL cholesterol, such as reverse cholesterol metabolism (reverse cholesterol transport), antioxidant cardiovascular protection, inhibition of platelet aggregation and anti-apoptotic and anti-inflammatory effects [58]. On the other hand, the alpha diversity of the microbiome in the Mexican child population tends to be higher in obese children than in normal weight children [28]. This could be related to altered diet and chronic low-grade inflammation, leading to an increase in microbial metabolites such as SCFAs. Although SCFAs are generally beneficial, in excess, they may contribute to obesity by increasing energy uptake by the host [52].

Recently, a study of 260 Mexican school-aged children from a socially disadvantaged population in San Luis Potosí, Mexico, was reported. This cohort showed that about 30% of the participants suffered from overweight or obesity and were randomly assigned to receive three types of diets enriched with some prebiotics, such as fructans from pineapple *Agave salmiana*, fermented milk (*Lactobacillus casei*), and inulin (a type of indigestible carbohydrate) for six weeks. These treatments did not result in significant differences in weight, height, BMI and body fat percentage, but the genera *Catenibacterium*, *Holdemanella*, *Agathobacter*, *Blautia*, and *Faecalibacterium* were most frequently found in the stool samples examined [51]. In a recent study of 330 Mexican children aged 6–12 years from Mexico City and Oaxaca (in the southwest of the country), they were categorized as normal weight or overweight/obese [61]. The authors reported that the *Bacillota/Bacteroidota* ratio showed a positive association with obesity, but this ratio showed a negative association with obesity depending on the locations where anthropometric measurements and biological samples were collected [61]. Specifically, in Oaxaca, the abundance of the phylum *Verrucomicrobia* (*Pseudomonadata* group) was negatively associated with obesity, while in Mexico City, the most common bacterial genera associated with obesity were *Ruminococcacea*, *Blautia*, *Fusicatenibacter*, *Intestinibacter*, *Ruminiclostridium*, *Clostridium*, *Anaerostipes*, and *Romboutsia* [42].

Méndez-Salazar et al. (2018) showed that both undernourished and obese children had significantly lower bacterial richness and diversity compared to normal weight children [33]. Undernourished children had a higher relative abundance of bacteria from the phylum *Bacillota*, especially from the family *Lachnospiraceae*, while the phylum *Proteobacteria* was overrepresented in obese children. Of note, the abundance of *Lachnospiraceae* was negatively correlated with total energy intake and positively correlated with circulating leptin levels. In addition, undernourished intake was found to be associated with specific microbial enterotypes: enterotype 1 (*Bacteroides*) was positively correlated with fat intake in overweight children and with carbohydrate intake in undernourished children. These results suggest that both diet quality and quantity contribute to shaping the gut microbial communities of children in extreme nutritional situations. Furthermore, higher dietary fiber intake has been associated with increased abundance of *Bacteroides*, a genus associated with favorable metabolic outcomes, while western dietary patterns rich in fat and simple sugars have been associated with increased prevalence of pro-inflammatory taxa such as *Erysipelotrichaceae*, which have been linked to MetS and obesity [26,33,62].

### 3.5. Studies of the Gut Microbiota in Adult Mexican Population with Obesity

Obesity is associated with reduced richness and diversity of the gut microbiota [12], but in the Mexican population, we found some discrepancies in terms of alpha and beta diversity which could be due to the relatively homogeneous diet in Mexico. This limited variation in diet could reduce differences in the diversity of the gut microbiota. In addition, the gut microbiota of children aged 5–10 years tends to resemble that of adults, contributing to the observed patterns: (a) García-Gamboa et al. reported that healthy Mexican adults did not show a significant reduction in alpha diversity compared to overweight and obese individuals [29], (b) Riggen-Bueno et al. reported that obese patients from western Mexico showed a significant reduction in alpha diversity compared to healthy individuals [40], (c) Chávez-Carbajal et al. found higher alpha diversity in Mexican women with obesity and MetS than in healthy women and women with obesity [26], and (d) Moreno-Altamirano et al. found that there were no statistically significant differences in alpha diversity between healthy, overweight and obese adults, including gender, BMI, socioeconomic status, and consumption of ultra-processed food [36]. The most abundant phyla were *Bacillota*, *Bacteroidota*, *Pseudomonadota*, and *Actinobacteroidota* [26,29,40]. At the family level, the *Lachnospiraceae* and *Christensenellaceae* were more common in healthy individuals [40]. The genera *Prevotella*, *Faecalibacterium*, *Histophilus*, *Rickenella*, *Odoribacter*, and *Marvinbryantia* were associated with normal weight individuals and showed a statistically significant positive correlation with anthropometric, biochemical, and nutritional parameters related to health and normal weight [29]. For example, *Prevotella* showed a negative correlation with BMI and carbohydrate intake, *Faecalibacterium* showed a positive correlation with high density lipoproteins (HDLs) and a negative correlation with fat mass and low-density cholesterol (LDL) [29]. *Histophilus* showed a positive correlation with HDL, but a negative correlation with BMI, weight gain, waist and hip circumference, carbohydrate intake, LDL cholesterol, and TG [29]. *Lachnospira* showed a positive correlation with TG, glucose, and very low-density lipoprotein (VLDL) [29]. *Odoribacter* showed a positive correlation with HDL and a negative correlation with BMI, WC, glucose and TG, as well as carbohydrate intake [29]. In contrast, obese and overweight individuals had a high number of bacteria that showed a statistically significant positive correlation with parameters associated with obesity. These bacteria were *Dialister*, *Phascolarctobacterium*, *Akkermansia* and *Subdoligranulum*, with the abundance of *Eggerthella*, *Allisonella*, *Subdoligranulum*, and *Dilema* being higher [29]. Of these, *Eggerthella* showed a positive correlation with LDL cholesterol, total cholesterol, and simple carbohydrate intake. *Allisonella* correlated positively with several anthropometric and metabolic parameters, including BMI, weight gain, biceps, subscapular and suprailiac skinfold thickness, waist and hip circumference, fat mass, VLDL cholesterol, TG, serum glucose, total carbohydrate intake, and energy intake. Similarly, *Clostridium* showed positive correlations with several parameters related to obesity, including weight gain, waist and hip circumference, BMI, fat mass, TG, glucose, VLDL cholesterol, carbohydrate and total calorie intake. In contrast, *Dielma* showed a positive association with body weight, BMI, triceps, subscapular and suprailiac skinfold thickness, fat mass, and energy and carbohydrate intake; however, these correlations did not reach statistical significance [29]. Regarding *Akkermansia muciniphila*, the greater abundance of *A. muciniphila* in obese and overweight individuals was associated with healthier lipid and glucose levels than in individuals with a lower abundance of *A. muciniphila* [29].

In the study of obese adults from western Mexico, a significant increase in the *Proteobacteria/Bacillota* (P/B) ratio was found, although the *Firmicutes*/*Bacteroidota* ratio showed no significant differences between the obese and control groups [40]. This study also found a significant increase in pro-inflammatory bacteria such as *Escherichia*/*Shigella*, *Prevotella*, and genera from the class *Negativicutes*. Of the class *Negativicutes*, the genera *Allisonella*, *Megamonas*, *Megasphaera*, *Acidaminococcus*, *Veillonella*, *Dialister*, and *Phascolarctobacterium* were reported to be absent in participants without obesity and, interestingly, have hardly been described in Latin American countries in the United States; therefore, their role in the pathophysiology of obesity is still unknown [40]; although both *Dialister* and *Phascolarctobacterium* have been reported to have a positive correlation with BMI and body weight gain, respectively [40] and that the pro-inflammatory potential of the *Negativicutes* class, which induces chronic low-grade inflammation, may reduce insulin sensitivity and increase lipogenesis in the liver [63].

The gut microbiota of healthy Mexican women (CO), women with obesity (OW), and women with obesity plus metabolic syndrome (OMS) is characterized by *Bacillota*, *Bacteroidota*, *Proteobacteria*, and *Actinobacteria* (from highest to lowest percentage of relative abundance) [26]. Several microbial signatures have also been identified that may underlie the metabolic differences between the groups. Six taxa, including the family *Lachnospiraceae* (associated with MetS), *Faecalibacterium* and *Megamonas* (associated with obesity), *Lachnospira*, *Coprococcus*, and *Ruminococcus* were significantly enriched in the OMS group. The CO group showed enrichment of seven taxa, including *Bacteroides*, *Streptococcus* (associated with body weight reduction and immunomodulatory properties), *Erysipelotrichaceae* (associated with metabolic disorders), *Parabacteroides*, *Staphylococcus* (associated with MetS), *Turicibacter* (with negative association with clinical indicators of metabolic disorders) and *Lactococcus*. In contrast, the OB group was characterized by increased levels of *Roseburia* (associated with obesity and type 2 diabetes), *Succinivibrio* and S24–7 (a lipopolysaccharide producer) [26]. The association analysis of clinical parameters with relative abundances in this population showed that the genus *Bilophila*—the more common genus in the OB group compared to the OMS group—had a positive association with body weight in all three groups [26].

The phyla *Bacillota* was most expressed in overweight and obese young adults, a finding related to an altered composition of the gut microbiota compared to normal weight subjects and the most significant microbial changes between normal weight and overweight/obese subjects were observed in *Clostridium coccoides–Eubacterium rectale* (phylum *Bacillota*) [41]. In another group of young adults, significant differences in gut microbiota were also observed between overweight and lean subjects, with *Bacillota* and *Bifidobacterium longum* being more abundant in the lean subjects, suggesting that a diet rich in unsaturated fatty acids and fiber promotes beneficial bacteria such as *B. longum* and *Bacteroidota* [31]. Interestingly, the genera *Dialister*, *Allisonella*, *Dielma* and *Roseburia* have been associated with obesity in other populations [64,65,66,67,68,69,70,71,72,73,74,75]. Although the genera *Phascolarctobacterium* and *Subdoligranulum* have been less extensively studied, their abundance has also been associated with obesity in some reports [36,63,76,77]. Of these genera, *Roseburia* has been studied in relation to obesity, as it is associated with higher fat intake, increased waist circumference, fat metabolism, fat storage and a pro-inflammatory profile in Mexican women with MetS, despite its beneficial SCFA production [78], as well as the benefits of some species such as *R. intestinales* that play an important role in regulating barrier homeostasis, immune cells, and cytokine release through its metabolite butyrate [79].

As mentioned above, there are few studies on the gut microbiota of obese adults in Mexico. Unfortunately, only two regions of the country have been studied, central and western Mexico, and as we show in Table 3, only three taxa overlap between these regions: *Prevotella*, *Lachnospira*, and *Clostridium* (Table 3).

### 3.6. The Role of the Traditional Mexican Diet in Obese Gut Microbiota

Diet and gut microbiota are factors for weight gain in obesity [80], and at the same time, diet is a factor that most influences the gut microbiota [81]. The diet of the Mexican population is geographically diverse; therefore, there are differences in the south, north, coast, mountains, and center of the country. For example, more meat is eaten in the north of the country, while vegetable consumption predominates in the south [82]. From a socio-cultural point of view, the “dieta de la milpa” is consumed in rural regions where food is produced locally and is hardly processed. This diet includes corn, beans, chili, red tomatoes and pumpkin seeds, chia, amaranth and peanuts, complemented by seasonal plants (quelites, nopal), a large number of local fresh fruits such as citrus and papaya, healthy fats such as avocado and moderate or small amounts of foods of animal origin [80,83]. However, in recent decades, younger age groups in rural areas have reduced their consumption of the dieta de la Milpa [84]. On the other hand, urban regions of the country have replaced the traditional diet with industrialized or ultra-processed foods, the so-called Western diet [61], which is low in fiber, high in saturated fat and simple carbohydrates [85]; therefore, there is a slightly higher tendency towards obesity in the north of the country and a lower one in southeast of Mexico [61,86].

In Mexican women with obesity in Mexico City who consumed 300 g of boiled nopal as part of a personalized diet plan, with an energy restriction of 500 kcal per day based on their predicted total energy expenditure, there was a decrease in the absolute frequency of families such as *Ruminococcaceae*, *Lachnospiraceae* and the genera *Prevotella* (*Prevotellaceae*) and *Ruminococcus* (*Lachnospiraceae*), *Streptococcus* (*Streptococcaceae*) and *Bifidobacterium* (*Bifidobacteriaceae*), while there were no statistically significant differences in alpha and beta diversity after the nopal diet [87].

In southern Mexico, a population from an urban region with overweight or obesity aged 18 to 25 years consumed less fruit, vegetables, cereals without fat, and fats without protein than the normal weight participants, with no differences in total energy (kcal) and macronutrients between the groups. In this population, microbial changes in *C. coccoides* and *E. rectale* were observed between groups. In the overweight and obese participants, fiber intake did not correlate with microbial variables. Interestingly, phylum *Bacillota* was most prevalent in the overweight and obese individuals, but the Kcal calculated from protein consumption correlated positively with the abundance of *Lactobacillus* in these groups [41].

In Mexican medical students, the differences in the gut microbiota were examined regarding fiber intake, consumption of ultra-processed foods, and BMI. In men, *Bacteroides* was more prevalent in overweight and obese participants. Alpha diversity showed no statistically significant differences between the groups studied. When the group was divided into three quartiles based on the energy content of ultra-processed food consumption, differences in beta diversity were found, but there were no differences between the BMI groups. Analysis of bacterial taxa for ultra-processed food consumption revealed a significant enrichment of *Prevotella* in subjects with below-average consumption compared to subjects with above-average consumption, while *Phascolarctobacterium* and *Streptococcus* were more abundant in subjects with above-average consumption [36].

In the urban Mexican child population aged 6 to 12 years, two dietary profiles were identified using a food frequency questionnaire: complex carbohydrates and proteins (diet 1) and saturated fats and simple carbohydrates (diet 2). In overweight and obese children, the *Bacillota/Bacteroidota* ratio and alpha diversity were higher, but not statistically different. Normal weight children showed an increased relative abundance of *B. rodentium*, *B. intestinalis*, *B. eggerthii*, and *Methanobrevibacter smithii*, while overweight and obese children showed an increased abundance of *Eubacterium* spp. and *Roseburia* spp. In normal weight children, the taxa *Bacteroides* appeared to increase with carbohydrate and fiber consumption and decrease with fat consumption, while the relative abundance of *M. smithii* decreased with protein intake. Among the taxa associated with overweight and obesity, *Roseburia* increased with the consumption of protein, fiber, and the overall healthier dietary profile 1, while the abundance of *Eubacterium* increased with sugar and saturated fat intake and decreased with fiber, polyunsaturated fat, and dietary profile 1. A greater relative abundance of *Eubacterium* spp. is associated with a higher BMI in children who also have a high intake of dietary profile 2. Therefore, proteins, simple and complex carbohydrates, and fats appear to be the main dietary factors responsible for the large differences in BMI and the impact on the microbiome in this population [88].

In another child population from Mexico City, overweight and obese children had a higher relative abundance of *L. reuteri* than children of normal weight. Children with obesity had a higher fructose intake and a higher proportion of fructose in their diet than children with normal weight and children with overweight. A high relative abundance of *L. reuteri* and a high proportion of fructose in the diet were directly and positively associated with greater adiposity [54]. Therefore, a traditional Mexican diet based on plant-based and unprocessed foods could promote a gut microbiota that supports the reduction in obesity [61,89].

## 4. Discussion

This systematic review aimed to investigate the bacterial composition of the gut microbiota of Mexican adults and children with overweight and obesity. Mexico is a country with a rich and distinctive heritage, characterized by a diverse, ethnically mixed population exposed to a variety of environmental factors and dietary habits that may contribute to the high prevalence of certain diseases, such as obesity and diabetes, which are now associated with microbiota dysbiosis. The study of the gut microbiota in obese Mexicans is a growing field, and although there is insufficient data from related studies, the studies included in this review show interesting diversity in the gut microbiota of obese adults and children.

There is increasing evidence that environmental factors such as diet and geographic location, as well as extrinsic factors such as economic status, play an important role in the composition of the gut microbiota in the Mexican population, particularly in children and adults [34]. Globalization and the rapid urbanization of developing countries such as Mexico have led to a change in dietary habits [90], moving away from the traditional foods of the Mexican diet. The transition from the “dieta de la milpa” to the most modern diet in the world—the Western diet—as well as lifestyle changes and genetic factors, have affected both school-aged children and adults, increasing the risk of obesity and chronic diseases [91]. Nevertheless, it is important to remember that while the traditional Mexican diet contains healthy elements, it also includes energy-dense foods and beverages that may not be consistent with current dietary guidelines [91].

Although there are no studies that have analyzed the gut microbiota in the northern states of Mexico, regional differences in microbial distribution have been observed, which may partly explain the disparities in metabolic outcomes. In studies comparing obese and normal weight children from Oaxaca, Querétaro and Mexico City, gut microbiota composition was associated not only with obesity status, but also with metabolic parameters such as TG levels, fasting plasma insulin [42] and HDL cholesterol [25], as well as pro-inflammatory (TNFa) and anti-inflammatory interleukins (IL-10) [86]. In particular, TG and insulin levels explained 6.8% and 7.8% of the variability in the gut microbiome of obese children from Oaxaca and Mexico City, respectively [42]. These results emphasize the influence of the metabolic status of the host on the ecology of the gut microbiome. A particularly interesting finding is that children from Oaxaca (81.2%) consumed a higher percentage of sugary drinks than children from Mexico City (78.7%) [42]. This result is remarkable considering that Oaxaca is predominantly rural while Mexico City is urban, which contradicts the general assumption that urban environments are more associated with the consumption of processed foods. This widespread consumption of sugar-sweetened beverages in both rural and urban populations in Mexico highlights a national nutritional problem that transcends geographical boundaries [3]. Considering that high consumption of added sugars is associated with insulin resistance, dyslipidemia, and increased obesity, the regional prevalence of sugary beverage consumption may contribute to the differences in metabolic profiles and microbiota composition observed in these populations, although in Oaxaca no genera were found to be associated with obesity status, while in Mexico City obesity status was positively associated with *Fusicatenibacter* and *Romboutsia* and negatively associated with *Ruminococcaceae* UCG-002 and *Ruminiclostridium* [42]. To complement the existing data from other regions of the country and to emphasize the significant role of diet in shaping microbial composition, it would be necessary to study the gut microbiota of obese people in northern Mexico. The northern states of Mexico have a significantly high obesity rate, making this population a high-risk group for metabolic diseases. Due to their proximity to the United States, these states tend to maintain a lifestyle more similar to that of the United States, especially in terms of dietary habits. This includes a high consumption of red meat (especially beef), dairy products, flour tortillas and processed foods—factors that are known to strongly influence the structure and function of the gut microbiota and consequently affect the health of the population in this region.

Specific microbial signatures have been associated with obesity in children from Mexico City. The genus *Bacteroides* is an important taxonomic group associated with obesity-related phenotypes in this population [23]. Among their species, *Bacteroides eggerthii* negatively correlated with BMI percentile and body fat percentage, an interesting finding that has never been reported in this population [23]. However, although *B. eggerthii* plays an important role in promoting obesity and inflammation, the mechanism underlying adiposity and weight gain remains unclear [17]. *B. plebeius* and members of the *Christensenellaceae* family were found more frequently in normal weight children than in obese children, but how they may influence BMI is also still unknown [23,92], underscoring a critical gap in our understanding of host–microbiome interactions in pediatric populations. Another compelling observation was the increased abundance of *Odoribacter* in obese children participating in a lifestyle intervention program [54]. This association suggests that *Odoribacter* may serve as a microbial marker for metabolic response or adaptation to lifestyle changes.

New microbial biomarkers were defined in children with obesity and MetS from Mexico City, which may also contribute to the understanding of the pathophysiology of these diseases. Specifically, increased relative abundances of members of the family *Coriobacteraceae* (*Collinsella* and *Collinsella aerofaciens*) and the family *Erysipelotrichaceae* (*Catenibacterium* and *Catenibacterium* spp.), the decreased abundance of *P. distasonis*, as well as the presence of *Porphyromonas* spp. and *Prevotella* enterotype were detected [28,31]. The above taxa are associated with a low-fiber diet (*Coriobacteriaceae* family, *Prevotella* spp.) [31,93], with host dyslipidemia associated with obesity, MetS and hypercholesterolemia (*Erysipelotrichaceae* family) [94], with the production of metabolites such as succinate and secondary bile acids that can reduce weight gain and hyperglycemia, improve glucose metabolism and alleviate obesity-related diseases such as liver disease (*P. distasonis*) [95] and obesity and type 2 diabetes, particularly in relation to periodontal disease (*Porphyromonas* spp.), suggesting a possible oral–gut axis contributing to a systemic metabolic disorder [96]. In the rural areas of Querétaro, the signature of the gut microbiota of obese children was similar to that of Mexico City, but with a lower proportion of *Bacteroidaceae–Porphyromonadaceae–Prevotellaceae* and a higher abundance of *Lactobacillaceae* [25]. In obesity, a decrease in the abundance of *Bacteroidaceae–Porphyromonadaceae–Prevotellaceae* appears to be associated with increased intestinal permeability, and thus with an increase in the absorption of lipopolysaccharide (LPS) [97]. The *Prevotella* enterotype in obese children showed different metabolic profiles compared to other enterotypes, and it is associated with a higher intake of hemicellulose (a component of dietary fiber), which is negatively correlated with markers of insulin resistance, suggesting a complex interaction between diet, gut microbiota composition, and metabolic health in this population, considering that Mexico City is an urban community [31].

The studies conducted in different regions of the state of Mexico, including Toluca, Chimalhuacán, and Ecatepec that surround Mexico City and are considered conurbated areas, have also defined different signatures of the gut microbiota. These regional studies emphasize the heterogeneity of the gut microbiome in response to nutritional and socioeconomic status. Obesity appears to be associated with an increase in specific genera related to energy production and metabolic inflammation, such as *Collinsella*, *Prevotella*, and *Roseburia*, while undernutrition may favor an overrepresentation of SCFA-producing *Bacillota* and *Proteobacteria*. In Toluca, obese children and adolescents showed a significant enrichment of members of the family *Coriobacteriaceae* (phylum *Actinobacteria*), with the genera *Lactobacillus* and *Coriobacteriaceae* being more abundant in children and the genera *Collinsell* and *Prevotella* in obese adolescents [38]. These microbial shifts suggest age-specific microbial adaptations or responses to obesity-related metabolic changes. The microbial signature of undernourished and obese children from low-income neighborhoods of Chimalhuacán consisted of a higher relative abundance of bacteria of the family *Lachnospiraceae* (phylum *Bacillota*) and the phylum *Proteobacteria*, respectively [33]. The *Lachnospiraceae* family is associated with increased energy yield; however, further studies are needed to determine whether the abundance of these bacteria in the first years of life predisposes undernourished children to obesity later in life. At the same time, the presence of *Proteobacteria* is a risk factor for human health, as they represent an imbalance in the gut microbial community and are a potential marker for disease risk [57]. These findings emphasize the complexity of microbiota composition, in which different phyla and families can be differentially modulated by malnutrition and obesity, as well as sociodemographic status. Studies on obese and overweight children from Ecatepec revealed elevated levels of *Faecalibacterium* spp., *Lachnospiraceae*, and *Roseburia* spp. [37]. These taxa are commonly associated with the fermentation of dietary fiber and the production of SCFA, suggesting that dietary patterns affecting these bacterial groups may play a role in the metabolic consequences of obesity. These microbiota profiles could provide valuable biomarkers for early detection and intervention strategies in pediatric nutritional disorders.

The composition of the gut microbiota of Mexican adults has only been determined in central and western Mexico and varies considerably according to body mass index, gender, geographic location and food intake, especially concerning fiber and unsaturated fatty acids. A hallmark of the obese microbiota was lower richness and diversity and increased abundance of pro-inflammatory taxa such as *Escherichia/Shigella*, *Prevotella* and members of the class *Negativicutes* in western Mexico [40,94] and a shift in the *Bacillota*/*Bacteroidota* and *Bacteroides/Prevotella* ratios in central Mexico [36]. Interestingly, a higher abundance of *Bacteroides* was observed in overweight and obese men, while *Prevotella* predominated in normal weight men; however, this trend was not observed in women, indicating possible gender-specific microbial responses [36]. The presence of beneficial taxa such as *F. prausnitzii*, *B. longum*, *C. leptum*, and *A. muciniphila* in individuals with a healthier metabolic profile [32] emphasizes the potentially modulating role of diet and microbial ecology in the treatment of obesity [29], as *F. prausnitzii* and *C. leptum* are both important butyrate producers that were less abundant in obese individuals [98], *B. longum*, a beneficial bacterium promoted by dietary fiber and unsaturated fats, supporting the protective role of diet on microbiota composition [29,32], and *A. muciniphila*, a mucus-degrading bacterium with known anti-inflammatory properties, which was reported to be more abundant in overweight and obese individuals who had a favorable lipid and glucose profile [99].

*Bacillota* were more abundant in women with obesity or metabolic syndrome (OB/OMS) from Mexico City compared to controls, consistent with previous reports of increased energy-harvesting capacity in obesity-associated microbiomes [26,100]. Significant changes were observed in *Ruminococcaceae*, *Lachnospiraceae*, and *Erysipelotrichaceae*. Interestingly, the *Erysipelotrichaceae* family was significantly reduced in OB/OMS individuals despite increased levels of beneficial taxa such as *Faecalibacterium* spp., *Roseburia* spp., and *Coprococcus* spp., highlighting the coexistence of both beneficial and pathogenic traits in obesity-related dysbiosis [26].

The regional studies from western Mexico have provided further insight into the composition of the gut microbiota in different BMI categories and revealed microbial changes that may precede or contribute to the development of obesity. In particular, higher levels of SCFA-producing *Lachnoclostridia* were observed in participants from the western states of Mexico, suggesting a potentially protective microbial function related to the fermentation of dietary fiber and regulation of host metabolism [40,41]. However, in young adults from Guadalajara, increased abundance of the *C. coccoides–E. rectale* group was found in overweight and obese individuals, suggesting an early shift in the microbiota that may lead to metabolic dysfunction. Other microbial profiles showed an enrichment of *Allisonella*, *Subdoligranulum*, *Dielma*, *Lachnospira*, *Romboutsia*, and *Clostridium* in obese individuals, while a higher abundance of *Flavonifractor*, *Eggerthella*, and *Alloprevotella* was found in overweight individuals. These genera have previously been associated with pro-inflammatory responses, altered bile acid metabolism and metabolic disorders, further supporting their potential role in the development of obesity. In contrast, normal weight individuals had greater proportions of *Faecalibacterium*, *Histophilus*, *Rikenella*, *Odoribacter*, and *Marvinbryantia*—genera commonly associated with anti-inflammatory properties, an intact gut barrier, and favorable metabolic outcomes [101]. These findings indicate that obesity in Mexican adults is associated with decreased diversity, increased pro-inflammatory and energy-harvesting bacteria, and different microbial profiles modulated by gender, diet, and geography. Fiber intake and a diet rich in unsaturated fats appear to be beneficial as they promote microbial taxa such as *B. longum* and *A. muciniphila*, which in turn has implications for therapeutic dietary strategies targeting the gut microbiota. The coexistence of beneficial and potentially pathogenic bacterial taxa in obese individuals suggests that metabolic disturbances mediated by the microbiota may trigger the early onset of obesity or exacerbate the obese state; therefore, early intervention in the gut microbiome through dietary or lifestyle changes may represent a promising avenue for the prevention of obesity in the Mexican population.

Finally, regional differences in the Mexican diet mean that people in rural areas adhere more closely to the traditional Mexican diet, which promotes a more diverse and metabolically favorable gut microbiota and lower insulin and cholesterol concentrations in Mexican adults [91]. Because the Mediterranean diet is associated with lower cardiovascular and metabolic risk [102], it is considered the healthiest alternative for reducing the risk of metabolic and cardiovascular disease. However, the components of the Mediterranean diet are expensive or difficult to access in certain regions of Mexico, making it difficult for many people to incorporate these foods into their daily meals [103]. In addition, the lack of affordability of healthy foods and the lack of time for physical activity are the main barriers cited by women, people with obesity, and people living in rural areas of Mexico [104].

The limitations of this review are as follows: (a) Only studies on the gut microbiota of children, adolescents, and adults from the central, southern, and eastern regions of Mexico are reported, (b) there are no reports of studies from the north of the country, (c) the food frequency questionnaire was not applied in 5 of the 19 studies included in this review, (d) the small sample size and low quality of some studies make their results somewhat unreliable, and (e) the lack of 16S rRNA sequencing in some studies limits the results of the taxonomic analysis of the gut microbiota.

## 5. Conclusions

In this review, we analyze the literature about the gut bacterial composition of the Mexican adults and children with obesity, highlighting the complexity and diversity of bacterial changes associated with this condition. In children, a broader and more diverse group of taxa was detected, including genera such as *Veillonella*, *Blautia*, *Alistipes*, *Holdemanella* and *Roseburia*, and families such as *Ruminococcaceae*, *Lachnospiraceae* and *Erysipelotrichaceae*; several species were also detected, including *Faecalibacterium* spp., *C. aerofaciens*, *P. distasonis*, *B. eggerthii*, and *B. longum*. In adults, obesity was primarily associated with significant changes in the relative abundance of taxa such as *Bacteroides*, *Prevotella*, *Escherichia/Shigella*, *Lachnoclostridium*, *Negativicutes*, *Bacillota*, *Bacteroidota*, and *Verrucomicrobia*. Despite the overlap of taxa between age groups, the microbial signatures of obesity in adults and children appear to differ in complexity and composition, possibly due to age-dependent interactions between host microbes, dietary habits, sociodemographic, environmental, and geographic factors, methodological approaches, and sampling protocols. The literature and data compilation here analyzed emphasize the complexity of gut microbiota–host interactions and highlight the importance of population-based studies. Therefore, the study of the microbiota in this context is a promising area that should be further explored, considering that Mexico has one of the most diverse plant-based diets in the world, which has a positive impact on the composition of the gut microbiota and may contribute to improved metabolic health and obesity treatment.

## Figures and Tables

**Table 2:** Overlap of taxa of the microbial signature of obese children in different regions of Mexico.

		Central Mexico			Eastern		Southern	References
		State of Mexico						
Bacteria	Mexico City	Chimalhuacán	Ecatepec	Toluca	Querétaro (Rural Areas)	San Luis Potosí	Oaxaca	
*Proteobacteria*	Present	Present						[33,35]
*Lachnospira* and *Roseburia*	Present	Present	Present					[33,37]
*Lactobacillus*		Present		Present	Present			[25,38]
*Faecalibacterium*			Present					[37]
*Bacteroides*	Present				Present		Present	[25,34,42]
*Porphyromonas*	Present				Present			[25,28]
*Family* *Ruminococcaceae*	Present							[42]
*Prevotella*	Present			Present	Present			[25,31,38]
*Collinsella*	Present			Present				[28,38]
*Alistipes*						Present	Present	[30,42]
*Family* *Coriobacteriaceae*	Present			Present				[28,38]
*Catenibacterium*	Present					Present		[28,30]

**Table 3:** Major taxa in the gut microbiota of obese adults in central and western Mexico.

Bacteria	Central Mexico (Mexico City)	Western Mexico (Colima, Jalisco, Michoacán, and Nayarit)	Reference
*Bacteroides*	Present		[36]
*Prevotella*	Present	Present	[36,40]
*Negativicutes*		Present	[40]
*Escherichia/Shigella*		Present	[40]
*Lachnoclostridium*		Present	[40]
*Allisonella*		Present	[29]
*Lachnospira*	Present	Present	[26,28]
*Romboutsia*		Present	[29]
*Subdoligranorum*		Present	[29]
*Clostridium*	Present (and *C. leptum*)	Present (and *C. coccoides*)	[29,32]
*Dilema*		Present	[29]
*Eubacterium rectale*		Present	[41]
*Faecalibacterium*	Present (and *F. praunitzii*)		[26,32]
*Roseburia*	Present		[26]
*Coprococcus*	Present		[26]
*Erysipelotrichaceae*	Present		[26]
*Bifidobacterium longum*	Present		[32]

## Data Availability

No new data were created or analyzed in this study. Data sharing is not applicable.

## Supplementary Materials

The following supporting information can be downloaded at: https://www.mdpi.com/article/10.3390/microorganisms13081727/s1, Table S1: Search strategies and results in the databases; Table S2: Quality assessment of cross sectional studies; Table S3: Quality assessment of quasi-experimental studies; Table S4: Quality assessment of randomized controlled trials.
