# Supplementary material for: The Bacterial Composition of the Gut Microbiota of Mexicans with Overweight and Obesity: A Systematic Review
Source: Microorganisms. 2025 Jul 24;13(8):1727. doi: 10.3390/microorganisms13081727 (PMC12388073; doi:10.3390/microorganisms13081727)
Supplement: Supplementary file 1 [file microorganisms-13-01727-s001.zip › microorganisms-3718659-supplementary.pdf]

Supplementary Table 1: Search strategies and results in the databases

Database	Search Strategy	Results
**Medline**	(obesity OR Adult obesity OR Childhood obesity OR Pediatric obesity)<br>AND<br>(human gut microbiota OR gut microbio OR microbio OR Gut bacteria OR Gut composition OR Intestinal bacteria OR Gut commensals)	**354**
**Web of Science**	(obesity OR Adult obesity OR Childhood obesity OR Pediatric obesity)<br>AND<br>(dietary patterns OR dietary interventions OR 1exican foods OR traditional 1exican diet OR 1exican diet OR Food consumption)<br>AND<br>(human gut microbiota OR gut microbio OR microbio OR Gut bacteria OR Gut composition OR Intestinal bacteria OR Gut commensals	**1899**
**CINAHLT**	TX Mexican humans with obesity OR TX Mexican adults OR TX 1exican children OR TX 1exican women OR TX rural community in 1exica OR TX 1exican schooled-children OR TX rural 1exica OR TX young 1exican adults<br>AND<br>TX obesity OR TX Adult obesity OR TX Childhood obesity OR TXPediatric obesity<br>AND<br>TX dietary patterns OR TX dietary interventions OR TX 1exican foods OR TX traditional 1exican diet OR TX 1exican diet OR TX Food consumption<br>AND<br>TX human gut microbiota or gut microbio*OR TX microbio*OR TX Gut bacteria OR TX Gut composition OR TX Intestinal bacteria OR TX Gut commensals	**9**
**Redalyc**	"mexican*" AND "obesidad" AND ("comida mexicana" OR "cocina mexicana" OR "dieta tradicional mexicana" OR "dieta mexicana" OR "consumo de alimentos")<br>AND<br>("microbiota intestinal humana" OR "microbiota intestinal" OR "bacteria intestinal" OR "composición intestinal" OR "comensales intestinales")	**29**
**SciELO**	obesidad AND microbiota intestinal AND México	**41**

Risk of bias assessment

Supplementary Table 2: Quality assessment of cross sectional studies

Number	Author	Criteria for inclusion clearly defined	Study subjects and the setting described in detail	Exposure measured in a valid and reliable way	Objective, standard criteria used for measurement of the condition	Confounding factors identified	Strategies to deal with confounding factors stated	Outcomes measured in a valid and reliable way	Appropriate statistical analysis used	Total score
1	Laura Moreno-Altamirano [36]	yes	yes	yes	yes	yes	yes	yes	yes	8
2	Verónica Riggen-Bueno [40]	yes	yes	yes	yes	yes	yes	yes	yes	8
3	Ricardo García-Gamboa [29]	yes	yes	yes	yes	yes	yes	yes	yes	8
4	Avilene Rodríguez-Lara [41]	yes	yes	yes	yes	no	no	yes	yes	6

7	Miguel Vázquez-Moreno [42]	yes	no	yes	yes	no	no	yes	yes	5
8	Tania Aguilar [25]	yes	yes	yes	yes	yes	yes	yes	yes	8
9	Luigui Gallardo-Becerra [28]	yes	yes	yes	yes	yes	yes	yes	yes	8
10	Sofía Morán-Ramos [34]	yes	yes	yes	yes	yes	yes	yes	yes	8
11	Alejandra Chávez-Carbajal [26]	yes	yes	yes	yes	yes	yes	yes	yes	8
12	Otoniel Maya-Lucas [31]	yes	yes	yes	yes	no	no	yes	yes	6
13	Khemlal Nirmalkar [38]	yes	yes	yes	yes	yes	yes	yes	yes	8
14	Blanca Estela Lopez-	yes	yes	yes	yes	yes	yes	yes	yes	8

	Contreras [23]									
15	Eder Orlando Méndez-Salazar [33]	yes	yes	yes	yes	yes	yes	yes	yes	8
16	Yaneth C Orbe-Orihuela [39]	yes	yes	yes	yes	yes	yes	yes	yes	8
17	Lino Mayorga Reyes [32]	yes	yes	yes	yes	yes	yes	yes	yes	8
18	Barbara Ixchel Estrada-Velasco [27]	yes	yes	yes	yes	yes	yes	yes	yes	8
19	S. Murugesan [37]	yes	yes	yes	yes	unclear	unclear	yes	yes	6

Supplementary Table 3: Quality assessment of quasi-experimental studies

Number	Author	Is it clear in the study what is the "cause" and what is the "effect" (i.e. there is no confusion about which variable comes first)?	Was there a control group?	Were participants included in any comparisons similar?	Were the participants included in any comparisons receiving similar treatment/care, other than the exposure or intervention of interest?	Were there multiple measurements of the outcome, both pre and post the intervention /exposure?	Were the outcomes of participants included in any comparisons measured in the same way?	Were outcomes measured in a reliable way?	Was follow-up complete and if not, were differences between groups in terms of their follow-up adequately described and analyzed?	Was appropriate statistical analysis used?	Total score
5	Sofía Morán-Ramos [35]	yes	no	not apply	not apply	yes	yes	yes	yes	yes	6

Supplementary Table 4: Quality assessment of randomized controlled trials

Number	Author	1. Was true randomization used for assignment of participants to treatment groups?	2. Was allocation to treatment groups concealed?	3. Were treatment groups similar at the baseline?	4. Were participants blind to treatment assignment?	5. Were those delivering the treatment blind to treatment assignment?	6. Were treatment groups treated identically other than the intervention of interest?	7. Were outcome assessors blind to treatment assignment?	8. Were outcomes measured in the same way for treatment groups?	9. Were outcomes measured in a reliable way	10. Was follow up complete and if not, were differences between groups in terms of their follow up adequately described and analysed?	11. Were participants analyzed in the groups to which they were randomized?	12. Was appropriate statistical analysis used?	13. Was the trial design appropriate and any deviations from the standard RCT design (individual randomization, parallel groups) accounted for in the conduct and analysis of the trial?	Total score
6	Marco U. Martinez-Martinez [30]	no	no	yes	unclear	unclear	unclear	unclear	yes	yes	no	yes	yes	yes	6
